# Current Demands for Food-Approved Liposome Nanoparticles in Food and Safety Sector

**DOI:** 10.3389/fmicb.2017.02398

**Published:** 2017-12-05

**Authors:** Shruti Shukla, Yuvaraj Haldorai, Seung Kyu Hwang, Vivek K. Bajpai, Yun Suk Huh, Young-Kyu Han

**Affiliations:** ^1^Department of Energy and Materials Engineering, Dongguk University, Seoul, South Korea; ^2^Department of Nanoscience and Technology, Bharathiar University, Coimbatore, India; ^3^Department of Biological Engineering, Biohybrid Systems Research Center (BSRC), World Class Smart Lab (WCSL), Inha University, Incheon, South Korea

**Keywords:** nanotechnology, liposomes, food, agriculture, nanosensors

## Abstract

Safety of food is a noteworthy issue for consumers and the food industry. A number of complex challenges associated with food engineering and food industries, including quality food production and safety of the food through effective and feasible means can be explained by nanotechnology. However, nanoparticles have unique physicochemical properties compared to normal macroparticles of the same composition and thus could interact with living system in surprising ways to induce toxicity. Further, few toxicological/safety assessments have been performed on nanoparticles, thereby necessitating further research on oral exposure risk prior to their application to food. Liposome nanoparticles are viewed as attractive novel materials by the food and medical industries. For example, nanoencapsulation of bioactive food compounds is an emerging application of nanotechnology. In several food industrial practices, liposome nanoparticles have been utilized to improve flavoring and nutritional properties of food, and they have been examined for their capacity to encapsulate natural metabolites that may help to protect the food from spoilage and degradation. This review focuses on ongoing advancements in the application of liposomes for food and pharma sector.

## Introduction

Food is a natural nano-structured substance. A simple step of boiling an egg can change its sub-nanometer level, as proteins tangle together to form a solid egg white. For decades, a number of processed foods have relied on processes that are now described as components of nanotechnology (Rashidi and Khosravi-Darani, 2011). For instance, tomato ketchup is composed of very small particles dispersed in the water, whereas the fatty grains of creamer coffee powder are coated with silica nanoparticles to prevent them sticking together. Current nanotechnology enables researchers to study what happens at this scale, and these studies can aid the design of new nanostructures that improve food quality (Kampers, 2016).

Nanotechnology combined with other technologies and scientific fields such as biotechnology, chemistry, physics, and engineering has increased transformative potential. Nanotechnology has shown several advantages in a variety of fields including, civil, mechanical, electronics and electrical engineering as well as new products for medicine, wastewater, potable water treatment, biology, biochemistry, agriculture, and food processing (Chellaram et al., 2014). In the food and agricultural fields, nanotechnology offers improved food security and processing, flavor, nutrition, delivery methods, pathogen detection, food functionality, environment protection, cost-effectiveness of storage and distribution, and improved abilities of plants to absorb nutrients (He and Hwang, 2016).

A longer time span of usability, insightful packaging, and more advantageous or functional-food nourishments containing dietary supplements are among the potential outcomes offered by nanotechnology. In 2016, the USFDA (US Food and Drug Administration) issued a draft directive on the utilization of nanotechnology in foods and nourishment related items (Food Safety Authority of Ireland [FSAI], 2008). The vulnerabilities associated with nanotechnology in foods are numerous, and the FDA requires venture owners to discuss them before marketing these types of products. This move has been welcomed by health and environment campaigners, as stated by George Kimbrell of the Campaign for Food Safety: “The agency is no longer ignoring scientific consensus that these nanomaterials have the capacity to be fundamentally different, and can create new and novel risks, necessitating new testing.” Nanotechnology is likewise seen by the food industry as a method for improving the food security and supplement bioavailability, and a few instances of these products are already being marketed in some countries (Food Safety Authority of Ireland [FSAI], 2008).

Liposomes have been utilized in immunoassays for decades in various formats supported by visible spectrophotometry. Liposomes are minor spheres extending in diameter from 50 nm to a few microns (Bangham and Horne, 1964) as well as vesicles composed of bilayers of phospholipids or other similar amphipathic lipids. Liposomes are used commercially as drug delivery systems, carriers for medical diagnostics, signal enhancers in analytical biochemistry, medicinal vehicles, solubilizers for various food ingredients to kill pathogens and detect toxins, and as penetration enhancers in the cosmetic industry (Papahadjopoulos, 1978; Kaes and Sackmann, 1991).

There are two types of liposomes, one called conventional and the other ones having modified surface properties. Conventional liposomes are first-generation products and include several lipid compositions with altered physicochemical properties. However, their biological properties do not change after intravenous administration (Umalkar et al., 2011). Liposomes tagged with bio-recognition agents such as DNA probe, aptamer, ganglioside, antibody, and streptavidin are used as signaling reagents for enzymes, fluorophores, latex beads, colloidal gold, and others (Edwards and Baeumner, 2013). The most commonly used recognition agents are antibodies, and antibody-sensitized liposomes (immunoliposomes) have produced encouraging results in various diagnostic applications. Immunoliposomes are prepared by tagging antibodies onto the outer surfaces of liposomes, which bind specifically to target antigens (Shin and Kim, 2008).

Immunoassays are biological analytical techniques in which the quantitation of an analyte relies on the specificity of the reaction between an antigen (analyte) and an antibody. Immunoassay methods are important pharmaceutical analytical tools for diagnosing diseases, detecting toxins, therapeutic drug monitoring, as well as clinical pharmacokinetics and bioequivalence studies in drug discovery and pharmaceutical development (Findlay et al., 2000). Immunoassays results have extremely high sensitivities and low limits of detection (Pieniaszek et al., 2003; Suzuki et al., 2003). Although antibodies and other signal generating labels are used for immunoassay development, antibodies are undoubtedly the key reagents (Darwish, 2006). Many types of signal generating labels are used in immunoassays, such as enzymes, fluorescent probes, metals and chelates, and liposomes. Based on review, the most common labels used in immunoassays are enzymes and liposomes due to their high signal amplifications and sensitivities. Immunoliposome-based immunoassays have been applied in food chemistry, clinical microbiology, food microbiology, nanobiotechnology, and diagnostics for the effective detection of food-borne pathogens, toxins, and hazardous elements in foods or the human body (Shukla et al., 2016c). Liposome immunoassays (LIA), which are based on enzyme immunoassays, utilize a liposome-encapsulated marker (SRB dye) prepared as phospholipid compositions coupled with either an analyte or antibody using standardized procedures (Shukla et al., 2012). Detection limits of these LIAs depend on liposome lysis as well as the release of encapsulated markers (Shukla et al., 2016c). Shin and Kim (2008) observed higher fluorescence signals as liposome size increased due to the greater number of SRB molecules encapsulated in larger liposomes, resulting in improved detection limits. Recent developments in immunoliposome assays include immunoliposome-coupled enzyme-linked immunosorbent assay (ELISA), immunoliposome-coupled magnetic separation, and liposome-based immunochromatographic strip assay (Shukla et al., 2016c).

Until now, there have only been a few studies published on the use of liposomes nanoparticles in various food systems. Further, the interactions of liposomes with food matrices are poorly understood with regards to potential food applications. Interest in liposome nanoparticles has increased due to their reported functional properties, including their physicochemical properties, kinetic stability, efficient encapsulation capacity, biocompatibility with food constituents, low cost of raw materials used for manufacturing, and their feasibility as nutraceuticals, antimicrobials, and flood flavoring agents (Hsieh et al., 2002; Laridi et al., 2003; Were et al., 2003; Taylor et al., 2005). The main advantage of liposome nanoparticles is their completely natural compositions, which can eliminate or even reduce issues related to their inclusion in various food systems (Taylor et al., 2005).

The main aim of this review is to provide updated information on the field of liposome nanoparticles from a research perspective relevant to food scientists. Information of liposome properties, manufacturing procedures, and their major applications in food and non-food systems such as medical systems and diagnostic strategies are discussed. The updated information and research strategies provided here may assist manufacturers and scientists dealing in food and/or food associated research making broader use of liposome-based promising technology in order to improve the quality and value of food products.

Additionally, we undertook the present review to provide an update on consumer requirements, the food industry in terms of safe food production, and approved legislation concerning the presence of hazardous toxins and food pathogens. In addition, common nanotechniques and liposome nanoparticle types, food safety concerns, and research progress are discussed.

## Nanostructures in Food

Food proteins are often globular structures with a size of 1–10 nm. Most polysaccharides (sugars) and lipids (fats) are direct polymers with sub-nanometer thickness, and the functionalities of numerous crude materials and the effective handling of food emerge from the presence, alteration, and production of self-assembled nano-structures (Chen et al., 2006; Ravichandran, 2010). For instance, gelatinization and benefits of nutritional influences are controlled by crystal structure of starch, starch-based processed food, and cellulose-fibril-based planer assemblies of plant cell wall, whereas fibrous structures control gel melting, setting, and texture. Oil–water and air–water interface originated two-dimensional (2D)-nanostructures control stabilities of food-foam and food-emulsion (Rudolph, 2004). Interpretation of chemical nature of food-based nanostructures can give new insights on selecting better raw materials that may enhance the quality of the food. Various analytical and innovative approaches such as atomic force microscopy, including electron microscopy have been used to determine the nature of these nanostructures (Chau et al., 2007; Ravichandran, 2010).

## Statistics Regarding Increasing Interest of Nanotechnology and Nanoparticles

According to statistics published on the StatNano website, ∼137,500 nanotechnology articles were indexed in the Web of Science (WoS) Database in 2016, which represents 9.5% of all articles indexed in the database. Of these 137,500 articles, 34% were published in China and 16% in the United States (Stat Nano, 2017). Nine of the 20 top countries that contributed are located in Asia and Oceania, and more than half of the nanotechnology articles were published in Asia and Oceania. Brazil was among top 20 Latin American countries with nanotechnology articles. Egypt was the top African country with 1,423 nanotechnology articles and ranked 25th, and South Africa was the second ranked African country (Stat Nano, 2017). In brief, in current years, China, United States, and India were ranked 1st, 2nd, and 3rd for publishing research in the related field of nanotechnology, thus showing increasing interest in the similar topics in the field of nanotechnology by the researchers of these countries.

**FIGURE 1:**
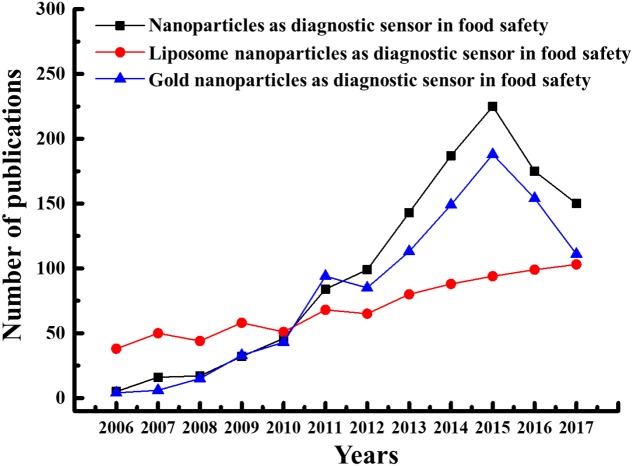
Number of publications in last 10 years in the area of nanoscience in food safety.

The number of publications on nanotechnology has soared during the last 10∼11 years (2006–2017), as depicted by the expanding number of scientific articles every year retrieved from the PubMed database by searching the term “nanotechnology,” “liposome,” “nanoparticles,” “gold nanoparticles,” and “food” in titles, abstracts, and keywords (**Figure 1**). Further, outputs probably underestimate the number of academic journal articles on nanotechnology topics unrelated to the food sector. As shown in **Figure 1**, the food safety sector has shown increasing interest in liposome nanoparticles, which can be used as alternatives for detecting various microbial pathogens and chemical contaminants in a variety of food materials and food products. As demonstrated, although the number of publications for gold nanoparticles (GNPs) are higher than liposome nanoparticles but decreased drastically after the year 2015, while in case of liposome nanoparticles, number of publications are also in increasing order after the year 2015 (**Figure 1**).

## Characteristics of Nanoparticles Useful in the Food Sector

Nanoparticles are invisible to the human eye. With a diameter of 100 nm or less, nanoparticles are 1,000 and 100-times smaller in diameter as compared to an average thickness of a book page and human hair, respectively. The various structures of nanoparticles can possibly be useful in different sectors of the food and pharmaceutical industries in food science-based research programs with proper processing and regulatory approval.

Nanoparticles incorporate huge range of classes associated with traditional materials, that include all types of metal, polymer, ceramic, and biomaterial. The development of ever smaller particles represents major challenges, mainly due to difficulties associated with characterizing the reliability, comparability, and reproducibility of materials on this scale. In light these challenges, a new sub-discipline of metrology is starting to evolve. Nanometrology, as it has become known, is the science of performing measurements at sub-100 nm dimensions as well as the characterization of these nanomaterials. However, without a righteous method of estimation, it is difficult to decide human or animal exposures via food or feed (Food Safety Authority of Ireland [FSAI], 2008). Of particular significance, any potential regulatory system requires assurance of the physiochemical properties of nanoparticles and estimation of their levels in food and feed. These properties incorporate size of the particle, distribution of the particle, surface range, surface charge, topography, purity and composition of particle, hydrophobicity, solubility, bioactivity, particle reactivity, and degrees of scattering.

## Mechanism of Liposome Formation

**FIGURE 2:**
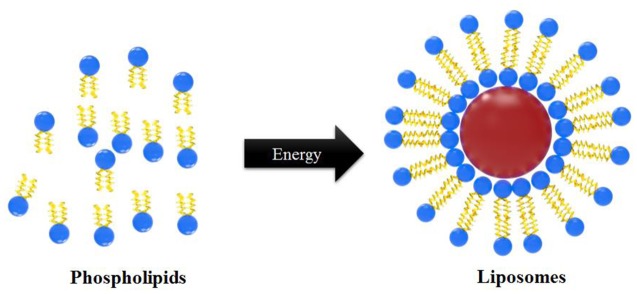
Mechanism of liposome particle formation.

The detailed mechanistic phenomenon of liposome formation is not yet well known. However, predominantly there are two distinct ways of vesicle formation in the lipid-in-water suspension, which include bilayer fragmentation with sub-sequent self-closure of bilayered fragments, and separation of sibling vesicle from parental liposome. In simple terms, when phospholipids are placed in water and sufficient energy is provided by sonication, heating, homogenization, or other methods, bilayer vesicles are formed (**Figure 2**) (Mozafari et al., 2008). This phenomenon is probably associated with the critical micelle concentration, defined as the concentration of lipids in water above which lipids form micelles or bilayer structures rather than remaining in solution as monomers (Sharma et al., 2010; Moghimipour and Handali, 2013) of phospholipids in water.

## Applications of Liposome Particles in Food, Agricultural, and Other Sectors

**FIGURE 3:**
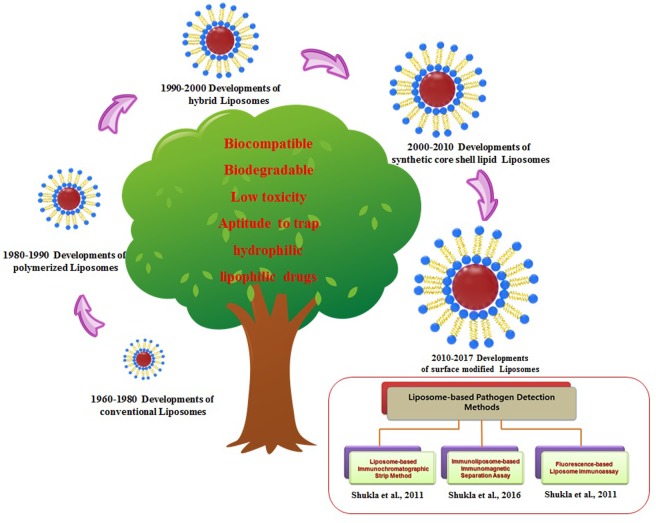
History and evolution of liposomes nanoparticles with applied strategies for the liposome-based detection methods.

Liposome-based micro-encapsulation techniques using edible biocompatible materials, including biopolymer matrices composed of sugar, starch, gum, protein, synthetics, dextrins, and alginates, are currently used in the food and medicine industries (Reineccius, 1995; Gibbs et al., 1999). Based on the successful use of liposome nanoparticles in biomedical and the pharmaceutical industries (cancer, and drug delivery, etc.), scientists have now started using liposomes for the controlled-delivery of functional constituents such as protein, polysaccharide, enzyme, vitamin, and flavor in a diverse range of food practices (Aishwarya et al., 2015). **Table 1** summarizes several commercialized liposome products that have been approved for different applications including drug development, food supplements and as food preservative. Nevertheless, despite their improved stabilities and enhanced circulation times, these polymer-stabilized liposomes lack selectivity for disease targets as well as controlled drug delivery. To overcome these limitations, several engineering strategies involving the modification of various liposome components have been employed, such as surface functionalization, to improve their performances and develop various liposome detection strategies (**Figure 3**). Few other recent developments and updates related to use of liposomes in vaccine development, and toxin detection are listed in **Table 2**. Further, there are very few food applications of liposome nanoparticles, which are discussed in the following sections.

**Table 1:** Approved selected liposome-based commercial products.

Liposomal product	Company	Role in human health	
Liposomal turmeric with fulvic acid	Purathrive	Anti-inflammatory	In dietary supplement
Liposomal curcumin syrup	Company Health	Anti-inflammatory	
Oral curcumin liposome syrup	Nutra Ingredients	Anti-inflammatory	
Liposomal vitamin C tablets	Dr. Mercola	Against vitamin C deficiency	
Liposomal glutathione capsules	Pure Encapsulations	Antioxidant support for liver	
Hemp liposomal syrup	Lipolife	Antioxidant, clinical strength	
Nisin-loaded liposomes	Various companies	For controlling spoilage and pathogenic bacteria in food	As a food preservative


**Table 2:** Updates on liposome nanoparticles used for vaccine developments and for detection techniques.

	Liposome type/formulation/test method	Target against	Reference
For vaccine developments	Lipopeptide vaccine	Group A *Streptococcus*	Ghaffar et al., 2016
	Mannosylated ionic liposome vaccine	HIV DNA vaccine	
	PEB modified liposome with CRX-601	Influenza vaccine	Oberoi et al., 2016
	Peptide-based liposome	Antitumor vaccine	Kakhi et al., 2015
	Liposome constituting microneedles	Oral mucosal vaccination	Zhen et al., 2015
	Liposome containing E-protein vaccine	Tembusu virus vaccine in duck	Ma et al., 2016
	Ophiopogon polysaccharide liposome	Parvovirus vaccine	Fan et al., 2016
For detection strategies	Liposome-encapsulated spin-trap (LEST)	Nitric oxide	Hirsh et al., 2016
	Liposome-based microcapillary immunosensor	*E. coli*	Ho et al., 2004
	GM incorporated liposome piezoelectric agglutination	Cholera toxin	Yu et al., 2011
	Secretory proteins encapsulated liposome-based detection	*Mycobacterium tuberculosis*	Tiwari et al., 2015
	Immunoliposomes-based assays	*Salmonella*, *Cronobacter*	Shukla et al., 2012, 2016c


## Liposome Nanoparticle-Encapsulated Enzymes in Dairy Foods

In recent decades, liposome nanoparticles have attracted huge interest for their use in the dairy food industry. During cheese ripening and hardening, time and costs can be reduced by the addition of edible enzymes such as proteinases prior to isolation of curds. However, addition of enzymes has some drawbacks such as rapid proteolysis of casein. As a solution to this problem, Law and King (1985) observed that addition of proteinases encapsulated in liposome nanoparticles to cheese-curd resulted in the formation of firm cheese through inhibiting the proteolytic process of β-casein and maintained the curd structure by preventing any enzymatic offense. Use of lipase for improving the production of cheese has been recently reviewed. Kheadr et al. (2002) reported that use of liposome-encapsulated lipase was able to reduce cheese firmness and increased the elasticity and cohesiveness of the cheddar cheese. In addition, addition of proteinases and lipases encapsulated in liposome nanoparticles to cheddar items should be attentively controlled for improved ripening and acceleration process in order to avoid negative effect on overall food quality. Composition of liposome is one of the key regulatory factors that must be considered by the manufacturers so as to keep the advancement of off-flavors and assure the payload release in a predictable way (Taylor et al., 2005).

### As a Food Fortifier

Liposome nanoparticles also have potential in the food fortification field. Due to low fish consumption in many countries, dietary supplementation with fish oil capsules appears to provide a straightforward means of increasing omega-3 LC PUFA intake (Kolanowski, 2010; Bender et al., 2014). Consumers have shown considerable interest in fortified foods containing different micronutrients (Siro et al., 2008). Nevertheless, the developmental challenges related to fish oil are substantial and include instability and undesirable flavors/odors. Further, their strong odors and rapid deterioration limit their applications in food formulations. Highly refined, odorless, or microencapsulated fish oil may provide a means of resolving undesirable sensory characteristics (Iafelice et al., 2008). For these reasons, Iafelice et al. (2008) used liposomes to nanoencapsulate fish oil for the fortification of yogurt. Ghorbanzade et al. (2017) also used liposome-encapsulated fish oil to fortify yogurt and observed that the sensory characteristics were closer to that of the control than yogurt fortified with free fish oil.

In recent years, several researchers have focused on encapsulation of various essential oils in liposome nanoparticles for overcoming the drawback of their being unstable and easy to degrade in natural environment since they can be easily affected by oxygen, light and temperature. Liposomes can protect the fluidity of essential oils and are stable at 4–5°C at least for 6 months (Sherry et al., 2013). Recently, Khosravi-Darani et al. (2016) reported increased antimicrobial activity of Zataria multiflora essential oil when encapsulated in liposomes. This can emphasize the potential use of these liposome nanoparticles while using natural products as potential conservative agents for using in the food and pharma industries. In addition, Sebaaly et al. (2015) synthesized clove essential oil (eugenol as a main bioactive compound) loaded liposomes, and found that these liposomes exhibited nanometric oligolamellar and spherical shaped vesicles and protected eugenol from degradation induced by UV exposure. Furthermore, these liposomes also maintained the DPPH-scavenging activity of free eugenol. These all studies proved liposome nanoparticles as a suitable system for encapsulation of volatile unstable essential oils for their various practical applicability.

In addition, liposome nanoparticles have been used for the fortification of dairy food products by using vitamins to enhance nutritional quality and shelf-life (Taylor et al., 2005). Banville et al. (2000) also observed a significant difference in the recovery of vitamin D from cheeses containing vitamin D encapsulated in liposome nanoparticles compared to commercially prepared vitamin D-containing cheese samples, confirming protection of vitamin D via liposomal encapsulation. Other dairy products have also shown increased use of liposome nanoparticles for inducing low rate of lactose digestion, which aids digestion of dairy foods by lactose intolerant β-galactosidase entrapped in soy-phosphatidylcholine (PC)/cholesterol liposome nanoparticles (Taylor et al., 2005). Hsieh et al. (2002) reported that liposome incorporating stearic acid and alpha-tocopherol were found effective in protecting entrapped alpha-amylase from pepsin assault, storage of cold temperature and outrageous pH condition.

## Liposome Nanoparticles for Stabilizing Food Components Against Degradation

In recent years, liposome nanoparticles have received interest for various food applications. Liposomes have been shown to preserve and protect vitamins in a variety of food practices. Previously, Kirby (1991) described that the antioxidant efficacy of liposome encapsulating ascorbic acid was dramatically increased when compared with free vitamin C solution. Liposome-encapsulated ascorbic acid retained >50% activity after several days of storage in the refrigerator, whereas free ascorbic acid lost all activity after 1 week of storage (Taylor et al., 2005). Additionally, other studies reported that the rates of light- or heat-induced degradation of retinol decreased upon encapsulation within liposomes regardless of the light source or temperature in comparison with free retinol (Lee S.C. et al., 2002). Further, Lee S.K. et al. (2002) also tested the antioxidant activity of free phosvitin and liposome-entrapped phosvitin in a model meat system (pork muscle homogenate and ground pork) and found that entrapped phosvitin was most effective than free phosvitin in inhibiting lipid oxidation in unsalted, uncooked port samples and least effective in inhibiting lipid oxidation in salted cooked pork samples (Lee S.K. et al., 2002).

Overall, above findings demonstrate potent efficacy of liposomes in food system to protect and maintain nutrient bioactivity from degradation in addition to the ability of liposome-entrapped vitamins to fortify the food in order to enhance overall food quality and health benefits.

## Encapsulation of Liposome Nanoparticles With Bacteriocin

Liposome nanoparticles have been utilized to encapsulate natural and synthetic bacteriocins. This represents an alternative approach to overcome the problems associated with direct application of bacteriocins to food products, such as proteolytic degradation or interactions with food components. Laridi et al. (2003) previously investigated the stability and effects of nisin Z entrapped in liposomes during cheddar cheese manufacturing. Although encapsulated nisin Z lowered the bacterial cell numbers of Lactococci, fermentation process of cheddar cheese formation was not severely hampered by encapsulated nisin Z, and it showed stability throughout the processing steps. In further support of the promising application of bacteriocin-loaded liposomes to cheese manufacturing (da Silva Malheiros et al., 2010). Benech et al. (2003) reported that nisin when encapsulated in the liposome had no adversary effect on proteolytic, rheological, and sensory properties of cheese, while direct incorporation of nisin producing strain into the cheese starter culture significantly altered proteolytic and lipolysis properties of cheese. However, no significant effect of nisinogenic strain was observed on rheological properties of cheese (da Silva Malheiros et al., 2010).

Further, da Silva Malheiros et al. (2010) encapsulated commercial nisin A into liposome nanoparticles made of partially purified PC of soybean as a cheap and readily available commodity. The prepared nisin-loaded liposomes presented high encapsulation efficiency and displayed enhanced antimicrobial activity. Overall, these investigations on liposome encapsulating antimicrobial peptides are advantageous in terms of comparing efficacy of liposome-entrapped molecule and free bacteriocin compound. These innovative research outcomes have encouraged storming demand on liposome-based research in order to improve food applications, shelf-life, and safety of food.

## Liposome Nanoparticles As Detection Sensor in Food Sector

### In Food Pathogen Detection

Kim et al. (2003) previously developed a procedure involving immunomagnetic separation as well as a test strip immunoliposome immunoassay for the sensitive detection of *Escherichia coli* O157:H7. Later, we tried to develop a rapid detection method for *Salmonella* spp. using an immunomagnetic separation/immunoliposome technique (Shin and Kim, 2008) and succeeded in developing a liposome immunosorbent immunoassay system using anti-*Salmonella* IgG (antibody)-tagged liposome (immunoliposome) to detect *Salmonella* Typhimurium (Shukla et al., 2012). More recently, we developed an immunoliposome-based chromatographic test strip assay for the detection of *S.* Typhimurium based on non-specific binding (Shukla et al., 2011). In these studies, immunoliposomes were developed by tagging with specific developed antibodies against the target pathogen. According to Chen et al. (2005), universal protein G-liposomal nanovesicles were easily conjugated by the antibodies within 30 min, and these conjugates (protein G-immunoliposomes) showed great detection ability against *E. coli* O157:H7 when incorporated in the immunomagnetic bead (IMB) assay. Further, Chen and Durst (2006) developed an array-based immunosorbent assay and confirmed the detection of *E. coli* O157:H7, *Salmonella* spp., and *Listeria monocytogenes* in pure and mixed cultures using protein G-liposomal nanovesicles. These findings confirmed the practical application of protein G-liposomal nanovesicles which can be used in various immunoassays to simultaneously detect foodborne pathogens as well as demonstrated their effectiveness as universal immunoassay reagents. Connelly et al. (2008) developed a bioanalytical detection method specific to viable human pathogenic *Cryptosporidium* spp. using lateral flow sandwich assays and liposome-encapsulated dye as a signal amplification system. These assays are inexpensive, rapid, specific, and straightforward to perform. Zaytseva et al. (2005) developed a microfluidic biosensor module based on DNA/RNA hybridization and liposome signal amplification using a fluorescence detector for the identification of pathogenic organisms and viruses. This biosensor module was designed for easy integration into micro total analysis systems that combine sample preparation and detection steps in a single chip. Park et al. (2004) also developed an immunoliposome sandwich fluorometric assay to detect *E. coli* O157:H7. These findings confirmed usefulness and practical application of immunoliposome-encapsulated fluorophores employing micro-titer plates in the automated and rapid detection of molecules at multivalent antigenic sites and demonstrated that immunoliposomes have wide applications for pathogen detection. Baeumner et al. (2003) also developed a nucleic acid coupled liposome-based RNA biosensor assay for the rapid detection of *E. coli* in drinking water. The developed biosensor, which used a membrane-based DNA and RNA hybridization system combined with a liposome amplification process, was shown to be specific for *E. coli* and did not produce false signals due to other microorganisms. Further, DeCory et al. (2005) developed an IMB-immunoliposome fluorescence assay for the rapid detection of *E. coli* O157:H7 in aqueous samples. Their results demonstrated the usefulness of immunoliposomes for detecting *E. coli* O157:H7 in aqueous sample incorporating IMBs and sulforhodamine B. Patricia and Durst (2000) also proposed the application of an LIA to the agricultural field. They developed a liposome immunomigration, liquid-phase competition strip immunoassay for the determination of potato glycoalkaloids. In this assay, polyclonal anti-solanine antibodies were raised and used. For this format, similar cross-reactivities were measured between the glycoalkaloids R-solanine and R-chaconine. This assay has been commercialized for the quantification of total glycoalkaloids in potatoes.

Previously, Zhao et al. (2006) reported liposome-doped nanocomposites as an artificial cell-based biosensor for the sensitive detection of a pore forming hemolysin, listeriolysin O (LLO) from bacterial origin. During pore formation and membrane insertion by LLO, immobilized liposome acted as cellular surrogates. Fluorescence quenching and leaching assays were used for measuring the integrity of liposomes in solid and solid-gel glass states. Although slower but a similar kinetics was observed by LLO in liposome-doped silica composites during pore formation. Further, insertion of LLO into immobilized-liposomes was found to be pH-dependent. Also, liposome doped composites did not show any increase in the membrane permeability in the presence of LLO at pH 7.4 (Gutierrez et al., 2009). In this study, immobilized liposomes detected LLO within ∼1.5 h and 30 min when steady state and kinetic calibrations were used, respectively. These liposome silica composites could potentially be used to detect hemolysin-producing *L. monocytogenes* and other bacteria that produce pore-forming toxins.

During the last decade, a variety of liposome-based assays have been developed to detect pathogens in various food matrices. Zhang et al. (2014) introduced a carbon-nanotube-based multiple-cycled liposome signal amplification assay for the detection of *E. coli* O157:H7 with a lowest detectable concentration (LOD) of 10^2^ CFU/mL. Bui et al. (2015) developed a liposome-amplified plasmonic immunoassay for the visual detection of single-digit, and live pathogens, including *Salmonella*, *Listeria*, and *E. coli* O157:H7, in water and food samples. This approach involved the integration of a cysteine-loaded nanoliposomes into a conventional ELISA as a signal amplifier as well as the release of cysteine from nanoliposomes to cause aggregation of plasmonic GNPs as a signal-amplified response. The lowest analyte concentration analyzed and detected with a visible color shift was found to be 6.7 attomolar, which is the lowest naked eye LOD reported without use of enzymes or visualization equipment. Although signal amplification approaches have shown highly significant potential for the detection of bacterial pathogens, still they face substantial challenges as far as their large-scale manufacturing repeatability is concerned. Thus, a simple, reproducible, and sensitive means of detecting low levels of *E. coli* O157:H7 is still required.

Rapid and cheap virus particle detection using liposomes is also a research topic of considerable interest. Reichert et al. (1995) developed sialic acid-conjugated liposomes that mimic cell surface molecular recognition and signal transduction for the colorimetric detection of influenza viruses. Virus recognition is based on binding between sialic acid and hemagglutinin lectin on the viral surface. Upon binding to influenza virus, liposome particles exhibited a color change from blue to pink/orange, which was quantitative spectroscopically (Shinde et al., 2012). Charych et al. (1993) previously optimized the colorimetric detection of influenza virus particles using polymerized liposomes containing sialic acid, and Charych et al. (1996) reported a LOD of ∼1 HAU in 250 μL (∼4,000 viruses per μL) using this method. Hidari et al. (2007) reported LODs as low as ∼0.1 pM (∼6 × 10^4^ viruses per μL) using a method based on surface plasmon resonance and immobilized sialic acid-containing liposomes. A similar LOD was obtained for influenza A virus using SPR based on a chip with immobilized bovine brain lipid-containing sialoglycolipids (Critchley and Dimmock, 2004). Egashira et al. (2008) developed a rapid and highly sensitive detection method by combining electrochemiluminescence with an immunoliposome-encapsulated Ru complex. Under optimum measurement conditions, hemagglutinin concentrations in influenza virus were measured in the concentration range of 3 × 10^-13^ to 4 × 10^-11^ g/mL, suggesting that 6 × 10^-19^ mol/50 μL could be considered as the LOD of this method for viral hemagglutinin. Notably, these findings showed that a method with high detection sensitivity at the attomolar level could be devised for detecting trace amounts of proteins in influenza virus. Damhorst et al. (2013) also developed an ELISA-inspired lab-on-a-chip strategy using an electrical sensing technique to detect biological entities tagged with liposome-encapsulated ion nanoparticles based on ion release impedance spectroscopy measurement. According to this method, ion-containing dipalmitoylphosphatidylcholine liposomes functionalized with antibodies were stable in deionized water but became permeable and released ions upon heating, suggesting that these liposomes could be optimal representatives for biosensing of surface-immobilized antigens electrically.

The authors further demonstrated the potential of these liposomes in quantifying viral counts using real-time impedance measurements as well as their feasibility as a reliable platform for the determination of viral loads with an ability to detect pathogenic microbes and other bio-fragments (Damhorst et al., 2013). In addition, these liposome-based methods for pathogen detection have been investigated by others (Shukla et al., 2016a,b; Song et al., 2016). Recently, attempts have been made to develop advanced liposome-based detection methods to achieve multiplexing and gain approval for commercial applications. Shukla et al. (2016a) reported limitations due to lack of multiplexing formats. However, since all liposome-based detection methods are based on fluorescence signals that differ according to various pathogenic bacteria, single-step multiple detection is a feasible target. Possible research result formats based on our earlier research were published by Shukla et al. (2016a,b).

### Pesticide Detection

A general awareness has become vibrant regarding extensive applications of pesticides and their deleterious impact on the global environment. Nowadays, there are huge applications of pesticides in food and agriculture industries including pest control. Although a trace amount of pesticide compound has ability to affect the central nervous system and can cause respiratory, myocardial, and neuromuscular malfunctioning. Further, due to their widespread use, pesticide residues can be found in the soil, atmosphere, agricultural products, and groundwater. For these reasons, methods are needed that can detect pesticides directly at low concentrations in foods and drinking water (Vamvakaki and Chaniotakis, 2007).

Pesticides are usually detected using analytical methods such as LC, GC (Hall et al., 1997), and MS (Yao et al., 1991). However, they require longer time for analysis, highly qualified technical hands for operation, and are highly expensive, and cannot be used in the field trials. Much effort has been expended to develop detection methods for pesticides, but few efforts have been made to develop pesticide detection methods based on liposome nanoparticles. Immunoliposome assays are used for various environmental monitoring purposes, including field determination of pesticides and other micro-pollutants in food and water samples.

Liposome-encapsulated ascorbic acid has been used in competitive assay formats (both lateral and horizontal flow formats) with amperometric detection following Triton X-100-induced lysis for the detection of the pesticide atrazine (Baumner and Schmid, 1998). Vamvakaki and Chaniotakis (2007) described a unique approach to the development of an AChE-based inhibitor nanobiosensor for pesticide analysis. A fluorescence detection scheme was chosen as a transduction scheme since it generally provides higher sensitivity, lower LODs, and wider detection ranges. The inherently unstable enzyme AChE from *Drosophila melanogaster* was previously encapsulated by liposomes, which showed great ability to improve enzyme stabilization against unfolding, denaturation, and dilution effect (Colletier et al., 2002; Chaize et al., 2003). Further, substrate release by liposomes has been shown to be facilitated by porins in the liposome membrane (Vamvakaki et al., 2005). In the method devised by Vamvakaki and Chaniotakis (2007), biosensor response was achieved using a pH-sensitive fluorescent indicator (pyranine; pKa 7.3) with a pKa close to the operational pH of AChE (pH 7.0). These stable nanobiosensors were used to directly detect of two widely used organophosphorus pesticides, dichlorvos and paraoxon, as well as to determine the total toxicities of drinking water samples.

### Toxin Detection

Wen et al. (2005) developed a novel method based on an Ara h1-tagged liposome-based lateral flow assay for the extraction and detection of peanut allergenic proteins in chocolate. Gangliosides, as natural toxin receptors of bacteria and viruses, are expressed on the surface of the cells, and several assays have been established for their specific targeting (Shinde et al., 2012). Singh et al. (2000) assembled GT1b or GM1 ganglioside-bearing liposomes to detect bacterial toxins of tetanus, botulinum, and cholera. These engineered liposomes acted as a cell and showed ability for the recognition of targeted toxins, thereby constituting a basis for the detection of specific toxins (Shinde et al., 2012). Further, a fluorescent rhodamine marker dye was used for the labeling of liposomes and a sandwich fluoroimmunoassay was applied on antibody-coated microtiter plate. Similarly, Ahn-Yoon et al. (2003) reported detection of cholera toxin employing GM1 ganglioside-containing liposome assay. In this bioassay, cholera toxin was detected as a colored band on nitrocellulose membrane strips, implying cholera toxin bound to liposomes was captured by immobilized antibodies. Ahn-Yoon et al. (2003, 2004) developed a ganglioside-LIA using the strip assay format for the ultrasensitive detection of cholera toxin and botulinum toxin and observed no any cross-reactivity. Ho and Wauchope (2002) developed a strip LIA for the detection of aflatoxin B1 at levels as low as 20 ng, thereby providing a potentially rapid means of visually screening agricultural and food samples. Hirsh et al. (2016) described a liposome-encapsulated spin-trap (LEST) method for the capture and *in situ* detection of NO (nitric oxide) by electron paramagnetic resonance and obtained a linear response for [NO] > 4 mM with a detection limit of [NO] > 40 nM in a 500 mL sample (>20 pmol). The LEST method determined time-dependent NO production kinetics with no inhibitory effect on the activity of inducible NO synthase or nitrate reductase. Also, the method showed minimal abiotic production of NO with nitrite and NADH. Further, the method could detect nitrate reductase-like activity in cell lysates of the coccolithophore *Emiliania huxleyi*, and this activity was shown to be elevated in virus-infected cultures. The LEST exhibited potential ability on the detection of NO in cell lysate and for the preparation of crude NO-producing tissue. Chu and Wen (2013) also described an advanced IMB-based liposomal fluorescence immunoassay for the detection of gliadin toxic and allergic protein in gluten-free food products. The assay incorporated anti-gliadin antibody-conjugated IMBs and fluorescent dye-loaded immunoliposomal nanovesicles (IMLNs) to capture gliadin in the sample and to produce and/or enhance the detection of the signals, respectively. The complex formed is an “IMB–gliadin–IMLN” sandwich. Based on the experimental results, the developed immunoassay exhibited good detection sensitivity of gliadin with a lowest gliadin detection limit of 0.6 μg/mL, although the polyclonal antibody used exhibited slight cross-reactivity with barley and rye.

A number of immunoassays utilizing antibody-coupled liposomes (immunoliposomes) were performed homo- and heterogeneously with an aim to detect antigens with multiple binding sites (Singh et al., 1995). In many of these homogeneous immunoassays, immunoliposomes are designed to undergo lysis upon exposure to targeted antigens that bind to antibodies on the surfaces of liposomes (Singh et al., 1995). On the other hand, most of the heterogeneous immunoassays devised with immunoliposomes use a sandwich format (Singh et al., 1995). Singh et al. (1995) utilized bifunctional vesicles tagged with enzymes and antibodies to detect *d*-dimer in human plasma and observed a nine-times lower detection limit than traditional ELISAs. Since the method employs increasing time of incubation to enable enzyme–substrate reactions for signal amplification and is thus unsuitable for routine automated assays. Rongen et al. (1995) developed a sandwich format of biotinylated liposome-encapsulated carboxyfluorescein employing a microtiter plate to measure the comparative sensitivities to that of the best colorimetric immunoassay for the detection of human interferon-γ. The assay system consisting a pump and auto-sampler was also automatized via placing a microtiter plate into a flow injection system. In the findings of Emanuel et al. (1996), it was found that polyethylene glycols steric barrier significantly improved the specific binding between immunoliposomes and targeted cells on the surface of the liposomes by preventing the non-specific binding on the surface of the cells. Currently, intensive efforts are being made on the use of immunodiagnostic assays employing these liposomes for the analysis of a wide range of analytes.

Research is also being conducted on other types of nanoparticle-based sensing devices for bacterial pathogens, virus particles, hazardous chemicals, toxic proteins, peptides, and other entities (Kim et al., 2016a,b). **Figure 3** represents the evolution of various kinds of liposome nanoparticles for their potential commercial usage. Our research group also has tried to develop applications and novel strategies for liposome-based detection of major food contaminants (**Figure 3**).

Liposome nanoparticles are noted down with several potent advantages, including low cost, easy biocompatibility and biodegradability, and easy surface modification nature of phospholipid bilayer as compared to conventional and other alternative nanoparticles such as GNPs, silver nanoparticles (SNPs), magnetic nanoparticles (MNPs), and silica nanoparticles (Shukla et al., 2011; Sung et al., 2016). Especially for diagnostic detections, liposomes can encapsulate hundreds of thousands of bioluminescence, fluorescent dyes or other signals which provide strong signal amplification. This makes liposomes very sensitive in biosensing assays as compared to other nanoparticles. Prior publications also show that liposome nanoparticles have received increasing research interest as diagnostic sensors due to their natural biocompatible properties than GNPs (**Figure 1**).

Although GNPs have proved as suitable candidates used for the diagnostic detection of foodborne pathogens, pesticides and toxins in food industries (Priyadarshini and Pradhan, 2017), apart from the positive factors, a majorly noted negative factor with liposome nanoparticles is associated with their low stability and shelf-life during their storage. Therefore, in order to improve the stability and long storage ability of liposome nanoparticles, researchers are finding the alternative ways to prepare stable liposome nanoparticles by re-constituting their composition. In a recent research, Peng et al. (2017) prepared hybrid liposomes, composed of amphiphilic chitosan and phospholipid and speculated that hydrophobic force between amphiphilic chitosan and phospholipid would help to stabilize liposomal membrane that may provide better detection strategies than GNPs and other detection strategies and may offer improved applications in food sectors.

## Pros and Cons of Industrial Applicability of Liposomes

Liposomes are composed of phospholipids, which in water form lipid bilayer spheres enclosing aqueous cores. Phospholipids are amphiphilic, and this characteristic is responsible for the formation of vesicles in aqueous solutions caused by hydrogen bonding, van der Waals forces, and other electrostatic interactions (Pattni et al., 2015). This vesicle structure means that liposomes can encapsulate hydrophilic or lipophilic molecules and determine the solubilities and *in vivo* fates of these encapsulated entities. The advantages offered by liposomes are as follows: (i) they improve the solubilities of encapsulated targets, (ii) they prevent the chemical and biological degradation of encapsulated materials under storage conditions, (iii) they reduce the side effects and toxicities for human health and thus improve efficacies and therapeutic indices, (iv) they can be chemically modified with specific surface ligands for targeting purposes, and (v) they are compatible with biodegradable and non-toxic materials.

According to a recent literature survey, liposome nanoparticles were used as a biological sensor for microbial pathogens, food toxins, and pesticides. Although a few nanobiosensors have been developed for various detection strategies, several other nanomaterials are also commonly used as an alternative to liposome nanoparticles for the detection of foodborne pathogens and toxins, including GNPs, gold nanorods, MNPs, quantum dots (QDs), SNPs, and silica nanoparticles (Leonard et al., 2003; Valdes et al., 2009; Lopez and Merkoci, 2011; Inbaraj and Chen, 2016). Generally, although the detection of foodborne pathogens and toxins is performed by exploiting the optical (optical sensors) or electronic (electrochemical sensors) properties of the nanomaterials as their value added characteristics (Lopez and Merkoci, 2011; Inbaraj and Chen, 2016), liposome nanoparticles have potential advantages over other kinds of nanoparticles used for various different approaches, including sensing approach because of their biocompatibility and biodegradability (Hsieh et al., 2002; Laridi et al., 2003; Were et al., 2003). Encapsulation or surface attachment of sensor materials as well as the subsequent release of liposome contents provide a simple and effective approach for signal amplification and transduction. Liposomes are compatible with current sensor technologies, including semiconductor QDs, nanoparticles, immunoassay, and electrochemical, fluorescence, and optical spectroscopy, etc. (Inbaraj and Chen, 2016). In addition, by incorporating different sensor materials and target molecules, liposome-based sensors can easily be modified for multi-target detection and sensing.

Previously, a number of different strategies of using GNP and SNP-based applications have been approved as potential alternatives in food industries for their use as detection biosensors, food fortifiers, enzyme and bacteriocin encapsulators and food stabilizers (Carbone et al., 2016; Fahim et al., 2016; Kang et al., 2016; Üzer et al., 2016). In spite of this, there are numerous other possible uses of liposomes in the food industries, including protection and stability of hypersensitive ingredients, and to improve the adequacy of food preservatives (Carbone et al., 2016; Fahim et al., 2016; Üzer et al., 2016). Encapsulation of liposome entrapped material has shown a wide range of chemical and environmental stabilities against chemical and enzymatic modifications, including temperature and pH stability. Also, implementation of any newly developed liposome-based formulation and complications on their regulation authorities can be promptly overcome because of their natural chemical composition. However, their successful commercialization is limited by liposome-associated cytotoxic effects. Further, many liposomes leak their payloads almost immediately after administration. In addition, it has been reported that charged liposomes are toxic (Alhajlan et al., 2013), and it is possible that some liposome production methods result in trace amounts of organic solvents in the final preparations. In addition, the storage stability of liposomes is a limiting factor in their application due to their low thermodynamic stability. Liposomes aggregate, fuse, and eventually precipitate overtime. Moreover, degradation could induce the release of encapsulated materials and shorten the shelf-life of liposomes during storage. Particle composition, pH, ionic strength, physicochemical properties of encapsulated materials, light, and environmental temperature can all reduce or alter the stabilities of liposomal products.

Liposomes also have some manufacturing-associated issues such as batch-to-batch irreproducibility, lack of effective sterilization methods, stability problems, and most importantly, scale-up problems. Classical methods such as use of transmembrane gradients as well as novel methods such as microfluidization, freeze-thawing, supercritical reverse phase evaporation, and spray-drying have been used to improve encapsulation efficiency and size reproducibility, and production of lyophilized liposomal powders for reconstitution can be used to improve stabilities. Further, no universal method has been devised for liposome sterilization, as filtration through 0.22 μm filters remains the most commonly used technique. However, this raises serious concerns regarding batch sizes and is perhaps the greatest concern regarding the scalability of liposome technology. Multifunctional liposomes also have production issues, which in combination with raw material costs means that liposomes tend to be expensive. Nevertheless, given the technological advancements made during the past decade, it is evident that liposome-based research developments will promote the use of liposome nanoparticles as effective detection and other food safety aspects.

## Conclusion

This review highlights the potential uses of liposomes and other nanomaterials for food analysis. Liposome-based sensors bind or react with the biological components of targets and generate detectable signals, which enable the rapid detection of food contaminants and enhance food safety by allowing prompt preventive action. In addition, they provide rapid, sensitive, and user-friendly detection assays that are portable and suitable for in-field applications. However, several issues such as interference during real-sample analysis, reproducibility, and toxicity remain to be resolved, and thus the need remains for more efficient liposome-based nanosensors for the food and medical sciences.

## Author Contributions

SS and VKB drafted and wrote the manuscript. SS, YSH, YH, SKH, and Y-KH contributed interpretation and analyzed data. VKB, YSH, and Y-KH contributed to conception and provided technical support.

## Conflict of Interest Statement

The authors declare that the research was conducted in the absence of any commercial or financial relationships that could be construed as a potential conflict of interest.
